# 

**Published:** 1914-10-03

**Authors:** 


### CONTENTS.

PAGE

**Physical Fitness and the Healing of Wounds** 1

**Unqualified Assistants for Infirmary Staffs ...** 2

**Hospital and Institutional News** ... ... ... 3

"The Hospital's" Special Number—Bedstead Lifters Competition— Hut Hospitals : A Red Cross Suggestion—New Secretary at North Ormesby Hospital—A Curious Public Misconception—Public Departments and Medical Etiquette—The Infirmary Question at Bath—A Doctor's Charges Against a Hospital—An Administrative Dilemma at Coventry—Panel Practitioners and Incapacity Due to Pregnancy—The New Pharmacopœia—Ulster Hospital for Service in France—Wives of Recruits and Maternity Benefit—Guardians and Military Camps—Army Airmen and the Westminster Guardians—Liverpool Dental Hospital and the War—The Recruits and Vaccination—The Obstruction of the Bromley Guardians—The Depleted Staffs of Glasgow Hospitals—The School of Pharmacy and the War—Women Pharmacists at London Infirmaries—Cardiff Hospital and Sir W. J. Thomas—Poor-Law Medical Officers and German Prisoners—The Success of a Central Drug Store—Grand Stand as a Hospital—New Treatment Baths at Bath—This Week's Drug Market.

**Types of Motor Ambulance Waggons** ... ... 9

The St. Andrew's Association's Models.

**The Transport of Wounded** ... ... ... ... 10

The Official Descriptive Statement by the Press Bureau.

PAGE

**Institutional Pharmacists and the War** ... 11

Conditions of Service of Army Compounders.

**Hospitals and the War** ... ... ... ... 12

**The Work of London and Provincial Hospitals**

Cardiff and District : Second Batch of Wounded Arrive — Chatham District : Anglo-German Hospital Train—Leicester : The Progress of the Wounded—Perth : New Infirmary—Sheffield : Second Convoy Arrive—Torquay : A "Palatial" Hospital — Tunbridge Wells : Emergency Work at Midnight—Wounded from the Aisne at the West Ham Hospital.

**Round the World** ... ... ... ... ... ... 14

Fellow-Workers and the Roll of Honour.

**Reports on Hospitals of the United Kingdom**— By SIR HENRY BURDETT, K.C.B., K.C.V.O. 15

The Royal Infirmary, Preston.

**National Insurance Act Developments** ... ... 17

New Government Grants.

**The Institutional Library** ... ... ... ... 19

**King Edward's Hospital Fund for London** ... 20

A Comparison of Hospital Expenditure in 1913 with 1912.

**The Editor's Letter-Box** ... ... ... ... 22

The British Pharmacopœia—The Cottage Hospital, Staines.

**The Institutional Worker** ... ... (Suppl.) 1

Fire Brigades and Hospitals.
The Care of Bedding.
Questions for October.

